# The impact of automated insulin delivery on glucose management in people with diabetes and advanced chronic kidney disease

**DOI:** 10.1007/s00125-026-06732-3

**Published:** 2026-05-11

**Authors:** Jean C. Lu, Christine L. Meyer-Olesen, Bella Halim, Sara Vogrin, Merete B. Christensen, John Apostolopoulos, Tobias Bomholt, Richard J. MacIsaac, Petrova Lee, Steven Trawley, Neale Cohen, Spiros Fourlanos, Stephen Stranks, Alicia J. Jenkins, Elif Ekinci, Mads Hornum, Kirsten Nørgaard, David N. O’Neal

**Affiliations:** 1 Department of Medicine, University of Melbourne, Parkville, VIC Australia; 2 Department of Endocrinology and Diabetes, St Vincent’s Hospital Melbourne, Fitzroy, VIC Australia; 3 Department of Nephrology and Endocrinology, Copenhagen University Hospital, Rigshospitalet, Copenhagen, Denmark; 4 Steno Diabetes Center Copenhagen, Herlev, Denmark; 5 The Australian Centre for Accelerating Diabetes Innovations, Melbourne, VIC Australia; 6 Department of Nephrology, St Vincent’s Hospital Melbourne, Fitzroy, VIC Australia; 7 Cairnmiller Institute, Hawthorn, VIC Australia; 8 University of Melbourne, Melbourne, VIC Australia; 9 Baker Heart and Diabetes Institute, Prahran, VIC Australia; 10 Department of Diabetes and Endocrinology, Royal Melbourne Hospital, Parkville, VIC Australia; 11 Southern Adelaide Diabetes and Endocrine Services, Adelaide, SA Australia; 12 University of Copenhagen, Copenhagen, Denmark

**Keywords:** Artificial pancreas, Automated insulin delivery, Diabetes complications, Diabetic kidney disease

## Abstract

**Aims/hypothesis:**

Chronic kidney disease (CKD) complicates insulin dosing and increases glycaemic instability in diabetes. We aimed to compare feasibility, safety and efficacy of automated insulin delivery (AID) with usual care in people with diabetes and advanced CKD.

**Methods:**

We conducted a prospective, open-label, randomised crossover trial at five tertiary hospitals in Australia and one tertiary centre in Denmark. Adults aged ≥18 years with type 1 diabetes or insulin-treated type 2 diabetes and advanced CKD (stage 3b or higher, including dialysis) were eligible. Participants were randomly assigned in a 1:1 sequence to receive either AID followed by usual care with real-time continuous glucose monitoring (CGM), or the reverse sequence, each for 8 weeks. Allocation was generated centrally using computerised randomisation. Due to the nature of the intervention, participants and clinicians were aware of treatment assignment. The primary outcome was percentage time in range (3.9–10.0 mmol/l) during the final 3 weeks of each treatment period.

**Results:**

Forty participants (24 type 1 diabetes, 16 type 2 diabetes; median [IQR] age 60 [55, 69] years; HbA_1c_ 64 [54, 73] mmol/mol [8.0% (7.1%, 8.8%)]; eGFR 30 [18, 37] ml/min per 1.73 m^2^) were enrolled: 33 not on dialysis, four on peritoneal dialysis and three on haemodialysis. AID significantly improved all hyperglycaemic CGM metrics compared with usual care. Time in range (3.9–10.0 mmol/l) improved from 60% (51%, 66%) at the end of usual care to 73% (65%, 78%) at the end of AID (*p*<0.001). Hypoglycaemia rates were unchanged. Participants were predominantly pre-frail at baseline and remained stable on-trial. No serious adverse events were attributed to the study devices. Nonetheless, 25% of participants experienced hospital admissions during the trial period for medical issues unrelated to device use.

**Conclusions/interpretation:**

AID is feasible and safe and compared with usual care provides superior glucose management in predominantly pre-frail people with diabetes complicated by advanced CKD.

**Trial registration:**

Australian New Zealand Clinical Trials Registry ACTRN12622000889752; ClinicalTrials.gov NCT06330194

**Funding:**

This trial was funded by the Australian Centre for Advancing Diabetes Innovations (ACADI), St Vincent’s Hospital Melbourne and Diabetes Australia.

**Graphical Abstract:**

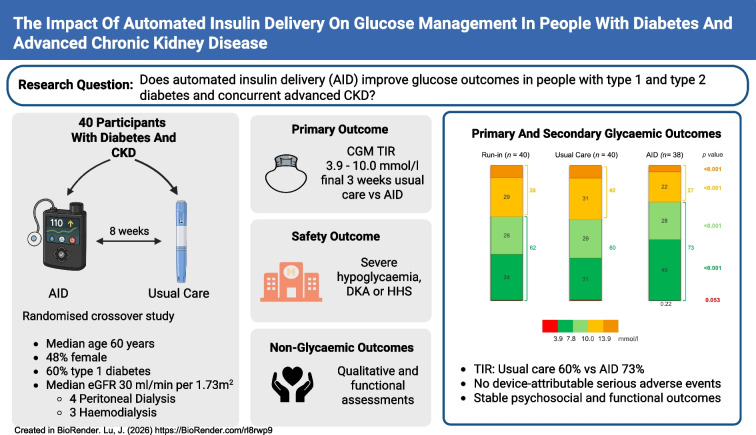

**Supplementary Information:**

The online version of this article (10.1007/s00125-026-06732-3) contains peer-reviewed but unedited supplementary material.


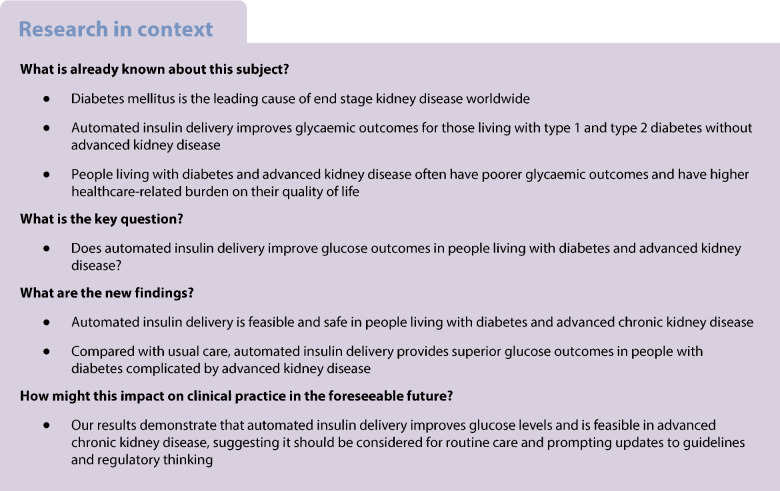


## Introduction

Diabetes is the leading cause of end stage kidney disease (ESKD) worldwide, accounting for approximately 22% of all ESKD cases in Europe [1] and 36% in Australia [2]. It represents a major global health challenge, consuming substantial healthcare resources [3–5]. Intensive glycaemic management is an established strategy to prevent the onset and progression of albuminuria and delay progression to ESKD [6, 7]. Moreover, increased glycaemic variability at the initiation of renal replacement therapy and during dialysis has been associated with reduced survival rate [8–11]. The coexistence of diabetic chronic kidney disease (CKD) and difficulty maintaining glycaemic targets is associated with increased morbidity, mortality and cardiovascular risk [12, 13].

Individuals with concurrent diabetes and advanced CKD (stage 3b or higher) often exhibit challenging glycaemic profiles and wide glycaemic excursions [14]. This phenomenon is multifactorial, driven by alterations in insulin and glucose metabolism that occur with advanced CKD, compounded by behavioural and cognitive factors [15]. Both peritoneal dialysis (PD) and haemodialysis (HD) introduce additional challenges: PD is commonly associated with hyperglycaemia and worsening metabolic syndrome, while HD is often complicated by frequent hypoglycaemia [16–18].

Automated insulin delivery (AID) systems have transformed diabetes management, particularly in type 1 diabetes. Clinical trials and real-world studies have consistently demonstrated improvements in glucose levels and enhanced quality of life [19–21]. Most commercially available AID systems require user-initiated meal announcements. While these systems provide dynamic and adaptable insulin delivery, users must still administer bolus doses to manage postprandial glucose excursions. Although these devices may be well suited to mitigate the marked glycaemic variability seen in advanced CKD, cognitive and physical limitations may be potential barriers to their use in an often frail cohort.

To date, research on AID in CKD has been limited. Existing studies suggest that AID can improve glycaemic outcomes in people with kidney failure; however, available data are largely limited to individuals with type 2 diabetes receiving maintenance HD and have primarily evaluated fully closed-loop systems that do not require meal bolusing [22–24]. More recently, a case series (*n*=9) demonstrated the feasibility and glycaemic benefits of various AID systems in people with type 1 diabetes and kidney failure on HD, highlighting the potential of this technology to address the substantial glycaemic variability associated with dialysis treatment [25]. Nevertheless, evidence remains limited regarding the use of hybrid closed-loop systems in broader populations with advanced CKD, including those not receiving dialysis, and with outcomes extending beyond glycaemic metrics. To address this gap, we conducted a randomised, crossover trial comparing AID therapy using the MiniMed 780G system with usual care over a 16 week period in free-living adults with type 1 diabetes or type 2 diabetes and concurrent advanced CKD. Outcomes assessed included glycaemic endpoints, psychosocial well-being, cognitive function and subjective sleep quality.

## Methods

### Study design

We conducted a prospective, open-label, two-stage, randomised crossover clinical trial (Australian New Zealand Clinical Trials Registry ACTRN12622000889752; ClinicalTrials.gov registration no. NCT06330194) comparing 8 weeks of AID with participants’ usual care plus real-time continuous glucose monitoring (CGM). The trial was conducted across five tertiary hospitals in Australia and one tertiary centre in Denmark. The original study protocol specified recruitment from six tertiary centres in Australia. One planned Australian site was subsequently unable to participate due to staffing constraints. To support recruitment and enhance representation of participants receiving dialysis, an additional site in Denmark was included after trial commencement. These modifications were made prior to completion of recruitment and did not affect eligibility criteria, study procedures or prespecified outcomes. The trial protocol was approved by a central Human Research Ethics Committee (HREC) (St Vincent’s Hospital Melbourne) and the Danish Medical Research Ethics Committee, with local governance provided at each participating centre. Written informed consent was obtained from all participants. All procedures were conducted in accordance with the ethical standards of the institutional and national research committees and with the principles of the Declaration of Helsinki [26].

The trial comprised eight study visits, including both a run-in and two intervention periods. Run-in lasted 4–6 weeks, with duration tailored to participants’ prior knowledge of carbohydrate counting and diabetes self-management. During this period, individualised education was provided by diabetes nurse educators and dietitians on topics including carbohydrate counting and use of an insulin bolus calculator. Following education, participants were trained in the use of CGM and underwent 3 weeks of unmasked CGM (Guardian 3 or Guardian 4) (Medtronic, Northridge, CA) to provide baseline data.

Following run-in, participants were allocated by an independent researcher in random order AID and their usual diabetes therapy in a 1:1 ratio for 8 weeks each. There was no washout period between the two phases. Randomisation was performed using a computer-generated minimisation algorithm, stratified by trial site and renal disease status (CKD stage 3b or greater, PD or HD).

Those assigned AID were trained in the use of the MiniMed 780G system (Medtronic, Northridge, CA), which consists of a glucose sensor (Guardian 3 or Guardian 4) with a Guardian Link transmitter, integrated with an insulin pump. To minimise risk in the study cohort the pump’s functionality was introduced stepwise:

- Weeks 1–2: Open-loop mode with predictive low-glucose suspend activated; glucose target set at 6.7 mmol/l
- Weeks 3–5: AID function enabled, with automated correction boluses disabled
- Weeks 6–8: Full AID function activated, including automated correction boluses; glucose target set at 6.7, 6.1 and 5.5 mmol/l at investigator discretion

Participants used their usual rapid-acting insulin (or insulin aspart if transitioning from mixed formulations). Meal-time boluses were participant-initiated, with estimated carbohydrate amounts entered.

For the usual care arm, participants continued with their standard insulin regimen (multiple daily injections [MDI], pre-mixed insulin injections or open-loop pump therapy) and oral hypoglycaemic agents as applicable. All wore the study CGM device (Guardian 3 or 4).

### Participants

Participants were recruited from tertiary diabetes and renal services at participating centres. The source population therefore comprised adults with type 1 or insulin-treated type 2 diabetes and advanced CKD receiving specialist care. As a result, the study cohort reflects a medically complex population typical of patients managed in tertiary diabetes–renal clinics but may not be fully representative of individuals with diabetes and CKD managed solely in primary or community care settings.

Demographic information collected included age and sex; additional information regarding ethnicity, socioeconomic status and regional background was not systematically collected. Participants were recruited in Australia and Denmark, providing representation from two high-income healthcare systems, although the cohort was predominantly of European ancestry. Where recorded, race or ethnicity was based on participant self-report. Sex was recorded for all participants based on self-report at the time of enrolment. Gender identity was not specifically collected. The study was not designed or powered to examine differences in outcomes by sex, and no prespecified sex-stratified analyses were performed.

Inclusion criteria were: adults aged 18–75 years with a confirmed diagnosis of type 1 diabetes of at least 1 year’s duration using MDI or a non-AID insulin pump, or those with type 2 diabetes requiring MDI or at least twice-daily pre-mixed insulin therapy; HbA_1c_ <91 mmol/mol (10.5%); impaired renal function defined as estimated glomerular filtration rate (eGFR, calculated by CKD-EPI creatinine equation [27]) <45 ml/min per 1.73 m^2^, including those with ESKD requiring PD or HD. Purposive sampling was applied to ensure that at least 40% of enrolled participants had either type 1 or type 2 diabetes.

Exclusion criteria included: current use of an AID-enabled insulin pump; treatment with oral hypoglycaemic agents other than metformin, GLP-1 receptor agonists or SGLT2 inhibitors (where permitted by regulatory guidelines); and systemic glucocorticoid therapy within the preceding month. Complete eligibility criteria are provided in electronic supplementary material (ESM) Table 1.

### Methods and outcomes

The primary outcome was the percentage of time CGM glucose values were within the target range of 3.9–10.0 mmol/l (time in range [TIR]) during the final 3 weeks of each study period. Secondary glucose outcomes included standardised CGM metrics for the overall 24 h period [28] and calculated Glycaemia Risk Index (GRI) [29]. Safety outcomes comprised the percentage of time spent with CGM glucose <3.0 mmol/l, hospital presentations for diabetic ketoacidosis or hyperosmolar hyperglycaemic state, and episodes of severe hypoglycaemia defined as an event associated with severe cognitive impairment requiring external assistance for recovery.

Non-glucose secondary outcomes included psychosocial, cognitive and functional measures: diabetes treatment satisfaction (Diabetes Treatment Satisfaction Questionnaire–Status [DTSQs]), health-related quality of life (EuroQol-5 Dimension [EQ-5D]), diabetes-related distress (Problem Areas in Diabetes [PAID] scale), fear of hypoglycaemia (Short Form Hypoglycaemia Fear Survey-II [HFS-II-10]), hypoglycaemia awareness (Gold Score and Clarke Score), cognitive function (Montreal Cognitive Assessment [MoCA]), sleep quality (Pittsburgh Sleep Quality Index [PSQI]), sarcopenia (Strength, Assistance in walking, Rising from a chair, Climbing stairs, and Falls [SARC-F]) and frailty (Fried frailty phenotype) [30–40].

### Statistical analysis

Sample size calculations were based on data from prior studies. Assuming a baseline time in target range of 55% with a standard deviation of 13.2% (derived from participants with type 1 diabetes and preserved renal function) and an anticipated increase to 69%, it was determined that 40 participants would provide >80% power to detect a 10% absolute difference between treatment arms, accounting for an estimated 10% dropout rate.

Baseline characteristics are presented as median (interquartile range [IQR]) and frequency (percentage). Primary analysis was on the intention to treat principle, including all stages with available data to the stage to which they were allocated, regardless of compliance to the intervention. All available CGM data were used. Sensitivity analysis included only stages where at least 70% of valid CGM readings were included [28]. The primary outcome was analysed by a mixed effects linear regression model using restricted maximum likelihood estimation with unstructured covariance. Participants were entered as random intercepts while intervention and period were entered as fixed effects. Results are presented as mean differences with 95% CIs (adjusted for period effect). All other continuous outcomes were analysed in the same manner. Some quality-of-life outcomes were transformed using the natural logarithm to enable better model fit (visual inspection of residuals); these results are presented as fold differences with 95% CIs. When, despite transformation, model fit was insufficient (hypoglycaemic and hyperglycaemic outcomes), the period-adjusted sign test (as described by Senn) was used [41]. These results are presented as median difference with 95% CI. Missing data were handled by a maximum likelihood approach within the model estimation. For non-parametric analysis, case-wise deletion was used. No adjustments for multiple comparisons among secondary outcomes were made. All *p* values were two-tailed; *p* values <0.05 were deemed to indicate statistical significance. Analyses were performed using Stata, version 18 (StataCorp, TX, USA).

## Results

**Fig. 1:**
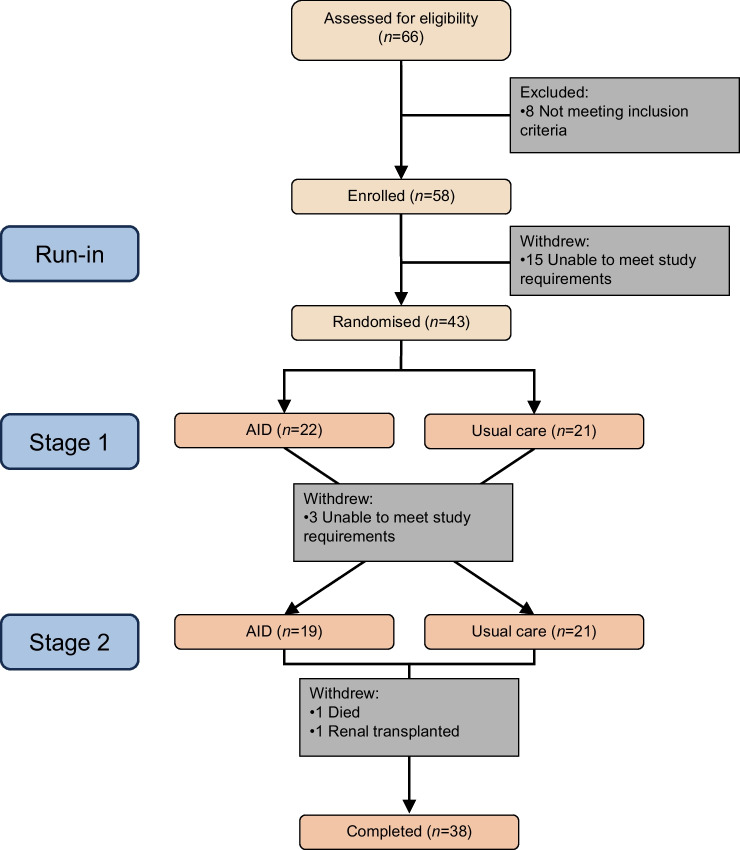
Participant flow through the study

Trial participants (*n*=66) were assessed for eligibility between 26 July 2022 and 31 January 2025 with eight failing screening, and 15 participants withdrew during the run-in period because they were unable to meet the demands of the trial protocol, particularly the requirement for frequent study visits and the volume and complexity of education and training delivered during the run-in phase. Between 6 September 2022 and 24 March 2025, a total of 43 participants were randomised. Three did not complete at least one arm of the trial. In total, 40 participants completed at least one arm of the trial and 38 of the randomised participants completed the full trial (Fig. 1). Nineteen (48%) of the participants were women. Participants’ ages ranged from 28 to 72 years and they had median diabetes duration of 28 (20–43) years. Twenty-four (60%) had type 1 diabetes and for all baseline HbA_1c_ at enrolment was 64 (54–73) mmol/mol (8.0% [7.1–8.8%]). Usual care was MDI for 35 participants (88%). Thirty-three (83%) were pre-dialysis individuals with eGFR <45 ml/min per 1.73 m^2^, four (10%) were on PD and three (8%) were on HD. The median baseline eGFR was 30 (18–37) ml/min per 1.73 m^2^ (Table 1).

**Table 1:** Baseline participant characteristics

Characteristic	Value
*n*	40
Age (years)	60 (55–69)
Sex	
Male	21 (53)
Female	19 (48)
BMI (kg/m^2^)	30.5 (27.1–35.0)
Diabetes type	
Type 1	24 (60)
Type 2	16 (40)
Diabetes duration (years)	28 (20–43)
Duration of insulin therapy (years)	23 (10–43)
Insulin delivery modality	
MDI	35 (88)
Insulin pump	5 (13)
HbA_1c_ at enrolment	
mmol/mol	64 (54–73)
%	8.0 (7.1–8.8)
Insulin TDD (U)	49 (33–86)
Insulin TDD/weight (U/kg)	0.6 (0.45–0.82)
Daily basal insulin (U)	25 (17–46)
Daily basal insulin/weight (U/kg)	0.31 (0.26–0.48)
eGFR at enrolment, ml/min per 1.73 m^2^	30 (18–37)
Previous CGM experience	25 (63)
Renal disease status	
Not on dialysis	33 (83)
PD	4 (10)
HD	3 (8)
Microvascular complications	
Diabetic kidney disease	37 (93)
Diabetic retinopathy	28 (70)
Peripheral neuropathy	16 (40)
Macrovascular complications	
Coronary artery disease	14 (35)
Cerebrovascular disease	6 (15)
Peripheral vascular disease	6 (15)
Frailty	
Robust	10 (25)
Pre-frail	27 (68)
Frail	3 (8)

Continuous data are presented as median (IQR). Categorical data are presented as frequency (%)

Percentages may not sum to 100 due to rounding

TDD, total daily insulin dose

### Glycaemic outcomes

**Fig. 2:**
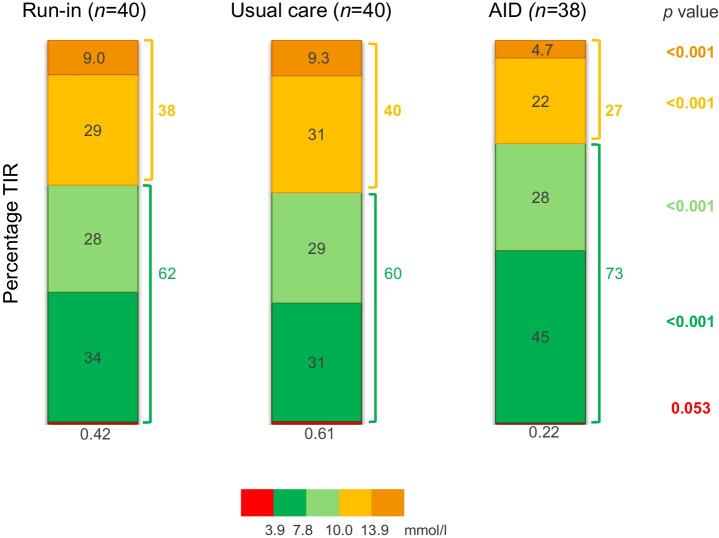
Primary and secondary glycaemic outcomes. Results are presented as median (IQR). The *p* values on study arms were calculated using the sign rank test adjusted for period effect. The colour key represents glucose concentration (mmol/l); values shown below the key indicate the glucose threshold at the transition between colours. Numbers to the right of each profile indicate CGM summary metrics: green values represent TIR (3.9–10.0 mmol/l) and yellow values represent time above range (>10.0 mmol/l), expressed as percentages and shown adjacent to the corresponding brackets. *p* values are colour coded to correspond to the respective glucose ranges within the bars; the light green *p* value corresponds to TIR and the yellow *p* value corresponds to TAR

In the primary analysis of the entire trial population (Table 2), the median (IQR) percentage TIR was 62% (50–79%) at baseline and increased from 60% (51–66%) at the end of usual care to 73% (65–78%) at the end of the AID period, representing a mean difference of 13.7% (95% CI 9.8%, 17.6%); *p*<0.001. All hyperglycaemic secondary glucose outcomes favoured the AID group with less time in high glucose ranges. Median percentage of time spent with glucose <3.9 mmol/l was 0.4% (0.1–1.3%) at baseline, 0.2% (0.05–0.6%) during AID therapy and 0.6% (0.1–1.1%) during usual care, yielding a treatment difference of −0.2% (95% CI −0.6%, 0.0%); *p*=0.053. Level 2 hypoglycaemia (<3.0 mmol/l) was very infrequent (0.05% [0–0.3%] at baseline) with no significant difference between the two treatment arms (Fig. 2). Sensitivity analyses limited to phases with at least 70% data completeness produced results consistent with the primary analysis (data not shown).

**Table 2:** Primary and secondary glucose and clinical outcomes

Outcome	Run-in (*n*=40)	Usual care (*n*=40)	AID (*n*=38)	Median difference AID vs control (95% CI)	*p* value
Percentage valid readings	90 (68–95)	94 (85–97)	96 (93–98)		
CGM% time 3.9–10.0 mmol/l	62 (50–79)	60 (51–66)	73 (65–78)	13.7 (9.8, 17.6)	**<0.001**
CGM% 3.9–7.8 mmol/l	34 (26–49)	31 (27–38)	45 (35–51)	11.8 (7.9, 15.6)	**<0.001**
CGM% time >10.0 mmol/l	38 (20–49)	40 (34–48)	27 (21–35)	−13.2 (−17.2, −9.2)	**<0.001**
CGM% time >13.9 mmol/l^a^	9.0 (2.2–14)	9.3 (5.2–16)	4.7 (2.3–7.1)	−5.4 (−6.9, −3.6)	**<0.001**
CGM% time >16.7 mmol/l^a^	2.1 (0.4–5.0)	2.8 (1.0–5.5)	1.1 (0.2–2.3)	−2.1 (−2.9, −0.3)	**<0.001**
CGM% time <3.9 mmol/l^a^	0.4 (0.1–1.3)	0.6 (0.1–1.1)	0.2 (0.1–0.6)	−0.1 (−0.6, 0.0)	0.053
CGM% time <3.3 mmol/l^a^	0.1 (0–0.3)	0.1 (0–0.3)	0.0 (0.0–0.1)	−0.0 (−0.1, 0.0)	0.127
CGM% time <3.0 mmol/l^a^	0.0 (0.0–0.1)	0.0 (0.0–0.1)	0.0 (0.0–0.0)	0.0 (−0.0, 0.0)	0.102
Standard deviation (mmol/l)	3.0 (2.5–3.5)	3.3 (2.7–3.7)	2.7 (2.3–3.0)	−0.6 (−0.7, −0.4)	**<0.001**
Mean glucose (mmol/l)	9.5 (8.1–10.0)	9.6 (9.2–10.0)	8.6 (8.3–9.3)	−1.1 (−1.4, −0.7)	**<0.001**
Coefficient of variation	33 (27–37)	33 (30–36)	31 (27–34)	−2.3 (−4.0, −0.7)	**0.005**
GRI	40 (22–51)	42 (34–53)	25 (22–35)	−17.6 (−22.4, −12.8)	**<0.001**
GRI classification (%)					
Zone A (green)	9	2	7		
Zone B (yellow)	11	17	26		
Zone C (orange)	14	16	5		
Zone D (red)	4	2	0		
Zone E (dark red)	2	3	0		
Insulin TDD (U)			42 (26–72)		
Insulin TDD/weight (U/kg)			0.5 (0.3–0.8)		

Results are presented as median (IQR). Difference between study arms presented as mean difference (95% CI), with *p* values obtained from mixed effects linear regression, unless specified otherwise

^a^Difference between study arms presented as median difference (95% CI). Analysis using sign rank test adjusting for period effect

TDD, total daily insulin dose

When outcomes were compared by diabetes type, glycaemic improvements with AID, while evident in both groups, were more pronounced in participants with type 1 diabetes than in those with type 2 diabetes. In the type 1 diabetes cohort, percentage TIR increased by 19.4% (15.1–23.7%) with AID vs usual care, compared with 5.3% (0.0–10.5%) in the type 2 diabetes cohort (*p* for interaction <0.001). Improvements in time above range were observed in participants with type 1 diabetes, whereas no statistically significant change was seen in the type 2 diabetes cohort. Mean glucose, standard deviation and GRI also showed greater improvement with AID in the type 1 diabetes group. Consistent with the full cohort analysis, hypoglycaemia of any level was infrequent and did not differ significantly between treatment arms, irrespective of diabetes type (Table 3).

**Table 3:** Primary and secondary glucose outcomes by type of diabetes

Factor	Type 1 diabetes					Type 2 diabetes					*p* interaction
	Run-in	Usual care	AID	Mean difference (95% CI)	*p* value	Run-in	Usual care	AID	Mean difference (95% CI)	*p* value	
*n*	24	24	23			16	16	15			
Percentage valid readings	88 (72–94)	93 (82–95)	95 (90–97)			93 (55–97)	97 (94–98)	98 (94–98)			
CGM% time 3.9–10.0 mmol/l	59 (52–76)	58 (50–65)	75 (68–79)	19.4 (15.1, 23.7)	<0.001	71 (46–80)	61 (53–72)	66 (59–77)	5.3 (0.0, 10.5)	0.049	<0.001
CGM% 3.9–7.8 mmol/l	33 (27–45)	31 (28–38)	45 (41–51)	15.8 (11.2, 20.5)	<0.001	42 (25–56)	31 (23–43)	41 (26–52)	5.7 (0.1, 11.4)	0.046	0.007
CGM% time >10.0 mmol/l	40 (20–48)	42 (34–49)	24 (21–32)	−18.8 (−23.2, −14.4)	<0.001	28 (19–54)	39 (27–46)	32 (23–41)	−5.0 (−10.4, 0.4)	0.071	<0.001
CGM% time >13.9 mmol/l^a^	9.7 (3.2–15)	11 (5.7–16)	3.4 (2.2–6.7)	−6.7 (−12.5, −3.9)	<0.001	6.1 (1.6–13)	8.4 (2.5–13)	6.1 (2.7–8.8)	−3.7 (−6.0, 0.3)	0.132	
CGM% time >16.7 mmol/l^a^	2.5 (0.7–5.9)	3.5 (1.1–6.2)	0.8 (0.2–2.1)	−2.5 (−4.8, −0.4)	0.001	1.5 (0.0–3.2)	2.1 (0.4–4.6)	1.3 (0.2–2.7)	−1.7 (−2.7, 0.0)	0.096	
CGM% time <3.9 mmol/l^a^	0.9 (0.3–1.8)	0.8 (0.4–1.2)	0.3 (0.1–0.7)	−0.4 (−0.7, 0.1)	0.102	0.2 (0.0–0.5)	0.1 (0.1–0.9)	0.1 (0.0–0.5)	−0.0 (−0.6, 0.0)	0.500	
CGM% time <3.3 mmol/l^a^	0.2 (0.0–0.7)	0.1 (0.0–0.5)	0.1 (0.0–0.2)	−0.0 (−0.3, 0.0)	0.049	0.0 (0.0–0.1)	0.0 (0.0–0.1)	0.0 (0.0–0.0)	0.0 (0.0, 0.0)	0.331	
CGM% time <3.0 mmol/l^a^	0.0 (0.0–0.3)	0.0 (0.0–0.2)	0.0 (0.0–0.0)	0.0 (−0.2, 0.0)	0.169	0.0 (0.0–0.0)	0.0 (0.0–0.0)	0.0 (0.0–0.0)	0.0 (0.0, 0.0)	0.323	
Standard deviation, mmol/l	3.2 (2.7–3.6)	3.5 (2.9–3.8)	2.6 (2.3–3.1)	−0.8 (−1.0, −0.5)	<0.001	2.8 (2.3–3.3)	2.9 (2.5–3.3)	2.9 (2.3–3)	−0.2 (−0.5, 0.0)	0.080	0.004
Mean glucose, mmol/l	9.6 (8.3–10)	9.7 (9.2–10)	8.5 (8.1–9)	−1.5 (−1.8, −1.1)	<0.001	8.8 (7.9–10)	9.5 (8.6–10)	9 (8.3–9.8)	−0.4 (−0.9, 0.1)	0.093	0.001
Coefficient of variation	34 (32–37)	35 (31–38)	31 (27–34)	−3.2 (−5.3, 1.1)	0.003	28 (25–36)	31 (28–33)	30 (25–34)	−1.1 (−3.6, 1.4)	0.397	0.217
GRI, median (IQR)	42 (33–51)	47 (37–56)	24 (20–32)	−24.6 (−29.8, −19.4)	<0.001	29 (18–51)	38 (26–51)	29 (23–38)	−7.4 (−13.8, −1.1)	0.022	<0.001
GRI classification											
Zone A (green)	3 (13%)	0 (0%)	5 (22%)			6 (38%)	2 (13%)	2 (13%)			
Zone B (yellow)	7 (29%)	9 (38%)	16 (70%)			4 (25%)	8 (50%)	10 (67%)			
Zone C (orange)	11 (46%)	12 (50%)	2 (9%)			3 (19%)	4 (25%)	3 (20%)			
Zone D (red)	2 (8%)	0 (0%)	0 (0%)			2 (13%)	2 (13%)	0 (0%)			
Zone E (dark red)	1 (4%)	3 (13%)	0 (0%)			1 (6%)	0 (0%)	0 (0%)			

Results are presented as median (IQR). Analysis by mixed effects linear regression (adjusted for period effect). Difference between study arms presented as mean difference (95% CI)

^a^Analysis by sign rank test (adjusted for period effect)

### Non-glycaemic outcomes

Overall, participants were predominantly pre-frail by the Fried frailty phenotype and demonstrated low risk of sarcopenia. This did not change. At baseline, participants reported mildly impaired health-related quality of life, with a median EQ-5D index score of 0.88 (0.73–0.97), which remained stable throughout the study (Table 4). Twenty-eight per cent and 23% of participants had impaired hypoglycaemia awareness as assessed by the Gold score and Clarke score, respectively; this did not change significantly over time. Fear of hypoglycaemia was minimal at baseline, as reflected by a median HFS-II-10 score of 7 (4–16), and remained unchanged during the trial. Diabetes-related emotional distress, measured using the PAID questionnaire, was low at baseline (23 [8–34]) and did not significantly vary across study periods. Treatment satisfaction was high at baseline (DTSQs score of 28 [26–31]) and improved following both intervention arms, with Diabetes Treatment Satisfaction Questionnaire–Change (DTSQc) scores of +16 and +12 for AID and usual care, respectively, although between arms this difference was not statistically significant. Sleep quality was poor at baseline (median PSQI 7 [4–12]) and did not differ between treatment periods. Cognitive performance, as assessed by the MoCA, indicated mild cognitive impairment in 16 participants (36%), while the remainder scored within the normal range. Scores remained stable with no significant change over the 6 month study period.

**Table 4:** Quality-of-life outcomes

Outcome	Baseline	End of run-in	AID	Usual care	AID vs usual care	
					Difference (95% CI)	*p* value
*n*	45	40	38	40		
EQ-5D EQ-VAS, median (IQR)	70 (50, 79) (*n*=42)	77 (62, 85) (*n*=35)	75 (68, 81) (*n*=33)	73 (62, 80) (*n*=38)	4.00 (−5.00, 10.00)	0.847
EQ-5D index, median (IQR)	0.88 (0.73, 0.97) (*n*=44)	0.83 (0.76, 0.97) (*n*=32)	0.92 (0.85, 0.97) (*n*=33)	0.93 (0.80, 0.97) (*n*=37)	0.00 (−0.03, 0.04)	0.060
Gold score, median (IQR)^a^	2 (1, 3)	2 (1, 3) (*n*=36)	2 (1, 4) (*n*=37)	2 (1, 3) (*n*=38)	0.37 (−0.06, 0.81)	0.095
Clarke score, median (IQR)^a^	1 (1, 3)	1 (0, 3) (*n*=37)	1 (0, 3) (*n*=36)	1 (1, 3) (*n*=38)	−0.07 (−0.44, 0.30)	0.707
HFS-II-10, median (IQR)^b^	7 (4, 16)	10 (3, 13) (*n*=35)	5 (2, 9) (*n*=37)	6 (2, 11) (*n*=38)	0.84 (0.67, 1.04)	0.107
PAID, median (IQR)^b^	23 (8, 34)	18 (5, 30) (*n*=37)	10 (3, 24) (*n*=36)	15 (3, 33) (*n*=38)	0.91 (0.71, 1.16)	0.435
PSQI, median (IQR)^b^	7 (4, 12) (*n*=44)	6 (4, 9) (*n*=36)	5 (4, 10) (*n*=37)	5 (3, 9) (*n*=38)	1.00 (0.89, 1.13)	0.986
MoCA, median (IQR)	26 (25, 28) (*n*=44)	27 (25, 29) (*n*=39)	28 (26, 29) (*n*=37)	27 (26, 28) (*n*=38)	0.00 (0.00, 1.00)	0.911
SARC-F, median (IQR)^a^		1 (0, 3) (*n*=35)	2 (0, 3) (*n*=37)	1 (0, 5) (*n*=38)	0.11 (−0.22, 0.43)	0.530
Fried frailty, median (IQR)		1 (1, 2) (*n*=40)	1 (1, 1) (*n*=38)	1 (0, 2) (*n*=39)	0.00 (0.00, 0.00)	>0.999
DTSQs median (IQR)	28 (26, 31)		32 (26, 36) (*n*=36)	30 (24, 32) (*n*=38)	1.00 (0.00, 4.00)	0.284
DTSQc, median (IQR)			16 (9, 18) (*n*=36)	12 (7, 15)	1.00 (0.00, 5.00)	0.559

For non-parametric tests (sign rank adjusted for period), results are presented as median difference (95% CI) unless otherwise stated

^a^Mixed effects linear regression used, with results presented as absolute mean difference (95% CI)

^b^Mixed effects linear regression used and outcome transformed using natural logarithm, with results presented as relative mean difference (95% CI)

EQ-VAS, EuroQol visual analogue scale

Safety outcomes were overseen by the HREC, with no trial-specific clinical interventions undertaken. A total of five serious adverse events (SAEs) occurred during the usual care phase and five during the AID phase (Table 5). One case of diabetic ketoacidosis was reported during the AID period, secondary to an acute myocardial infarction, and was deemed unrelated to the study device. A single episode of severe hypoglycaemia occurred in the usual care arm, requiring emergency department presentation. All remaining SAEs were hospital admissions for medical events unrelated to diabetes, including three admissions for falls complicated by femoral fracture, myocardial infarction and cardiac arrest. Additional events comprised two acute cardiac admissions, one episode of acute pancreatitis and one severe non-anaphylactic reaction to an iron infusion. An additional participant experienced a cardiac arrest and died during the study. No SAEs were attributed to the AID device.

**Table 5:** Safety outcomes: SAEs during randomised period

Outcome	AID *n*=40	Usual care *n*=40
Any SAE		
Number of events	5	5
Number of participants (%)	5 (12.5)	5 (12.5)
Severe hypoglycaemia		
Number of events	0	1
Number of events related to study device	0	–
Number of participants (%)	0 (0)	1 (2.5)
Diabetic ketoacidosis		
Number of events	1	0
Number of events related to study device	0	0
Number of participants (%)	1 (2.5)	0 (0)
Any other untoward medical occurrence		
Number of events	4	4
Number of events related to study device	0	0
Number of participants (%)	4 (10)	4 (10)

–, not applicable

## Discussion

We report a randomised crossover trial evaluating a commercially available AID system in individuals with advanced CKD and either type 1 or type 2 diabetes in a free-living environment. Over the 8 week intervention, AID use improved TIR by 13.7% compared with usual care plus CGM. This corresponds to approximately 3.3 additional hours per day within the target glucose range for participants using AID. The improvement in TIR was primarily driven by reductions in time above range, with AID significantly improving all hyperglycaemia-related CGM metrics in the intervention period. The overall occurrence of hypoglycaemia was low across all time points, probably reflecting the consistent use of CGM and diabetes-related education throughout the study among all participants.

The improvements in glycaemic parameters were more marked in the type 1 diabetes cohort; however, AID also conferred clinically meaningful benefits in those with type 2 diabetes, with increases of over 5% in both TIR and time-in-tight-range (3.9–7.8 mmol/l). The greater magnitude of improvement observed in type 1 diabetes probably reflects fundamental differences in disease pathophysiology, including absolute insulin deficiency and greater baseline glycaemic variability, which render a greater relative benefit in this population to AID algorithms. In contrast, individuals with type 2 diabetes often have a higher burden of comorbid illness, more insulin resistance and less prior experience with intensive insulin therapy and diabetes technologies, factors that may attenuate the relative impact of AID despite clinically meaningful gains.

The study population was older (median age 60 years) and characterised by low baseline technology fluency, borderline cognitive function, a high comorbidity burden and diminished strength, all of which contributed to challenges in meeting study requirements amid frequent medical appointments and competing health-related demands. Although analysis of AID downloads demonstrated sustained CGM use and stable activation of AID, it should be acknowledged that this occurred in the context of intensive education and close clinical support. Even within the structured environment of a clinical trial, this cohort posed significant challenges to implementation, reflected by the relatively high proportion of participants who failed screening or withdrew from the study. Nevertheless, with appropriate guidance and assistance, most participants (including two who were legally blind, one of whom had no prior experience with insulin pump therapy) were able to use AID safely and derive meaningful glycaemic benefit. Notably, 25% of participants experienced a serious event requiring hospitalisation during this short-duration study, underscoring the substantial disease burden of the cohort. Objective assessments of frailty and sarcopenia may therefore have underestimated the true vulnerability of this population, which was more accurately reflected by the frequency of non-device-related SAEs, including one death, reinforcing both the feasibility and the practical challenges of implementing AID in medically complex, compromised adults with advanced CKD and the need for structured education and flexible clinical support, with a simplified user interface to support broader adoption.

Only five participants had prior insulin pump experience, representative of the broader advanced CKD and diabetes population, who often face socioeconomic disadvantage, lower health literacy and engagement, and limited access to private insurance and diabetes technology [42]. The usual care arm, which included CGM provision, reflects the glucose management support typically available to most adults with type 1 diabetes, but substantially exceeds that which is generally accessible to people with type 2 diabetes, for whom self-monitoring of blood glucose remains standard care. Therefore, our results may underestimate the glycaemic impact of the intervention in free-living adults with type 2 diabetes. Structured run-in education covering carbohydrate counting, self-management skills and prandial insulin adjustment ensured comparable capability across groups; however, this education may have improved glycaemic literacy in participants prior to baseline, potentially attenuating the apparent treatment effect of AID compared with usual care.

Participants in this study reported stable psychosocial and functional outcomes throughout the trial. Health-related quality of life, measured by the EQ-5D index, was mildly impaired at baseline and unchanged with either intervention, consistent with prior reports that quality of life in CKD is influenced by multiple factors including socioeconomic status and comorbidities rather than short-term changes in glycaemic management [43]. Fear of hypoglycaemia and diabetes-related emotional distress were both low at baseline and remained stable, suggesting psychological safety and good adaptation to AID use.

Treatment satisfaction was high at baseline and improved following both study arms. The provision of CGM throughout the study and comprehensive diabetes education, including carbohydrate counting and individualised insulin adjustment, probably enhanced participants’ understanding of their diabetes management and contributed to improved treatment satisfaction and confidence in glycaemic management even in the usual care arm. The lack of a significant increment in treatment satisfaction following AID use may reflect a balance between the potential benefits of AID and the additional burden associated with integrating new technology into daily diabetes self-management and meeting trial protocol requirements. Self-reported sleep quality was poor at baseline but was not adversely affected by AID use, despite previous reports suggesting a potential negative impact of AID systems on sleep [44]. Devices optimised for those with physical and cognitive limitations and longer-term studies may lead to improvement in patient-reported outcomes. Further studies are warranted.

This study has several notable strengths. It is among the first to evaluate the use of AID in adults with advanced CKD in a free-living environment. This is a population that has historically been under-represented in diabetes technology trials. The crossover design minimised between-participant variability and strengthened the internal validity of treatment comparisons. Comprehensive assessment of psychosocial and functional outcomes, including quality of life, hypoglycaemia awareness, emotional distress, sleep quality, cognition and frailty, provided a multidimensional evaluation of intervention effects beyond glycaemic endpoints. The inclusion of CGM and structured diabetes education, incorporating carbohydrate counting and individualised insulin adjustment, ensured participants were well supported and able to engage effectively with the technology. Centralised data collection and close clinical supervision further enhance the reliability and interpretability of findings.

An important consideration in our study is the use of two different CGM systems, Guardian 3 and Guardian 4. This variation arose due to changes in CGM availability within the Australian market over the course of the study, reflecting the dynamic nature of diabetes technology access in real-world clinical practice. Importantly, each participant completed the entire study using the same CGM system across both the intervention and control periods, ensuring that within-person comparisons were not affected by differences in sensor performance.

There were several other limitations to the study. It was conducted at tertiary centres, which may limit generalisability to broader clinical settings where access to multidisciplinary diabetes care and diabetes technology education is less comprehensive. The study population was heterogeneous, comprising individuals with type 1 and type 2 diabetes across a spectrum of advanced CKD, including pre-dialysis, PD and HD. While this diversity reflected the heterogeneity observed in those with diabetes and kidney disease and enhances real-world relevance, it also resulted in small numbers within individual subgroups, limiting statistical power for subgroup analyses. In particular, the small number of participants receiving dialysis limits conclusions regarding the applicability of AID in this subgroup, as individuals undergoing HD or PD may experience greater physiological instability and competing treatment demands that could attenuate the magnitude of benefit achievable with AID compared with those with advanced CKD not yet receiving dialysis.

The study cohort included a broadly balanced distribution of male and female participants; however, the study was not designed or powered to detect sex-specific differences in outcomes, and sex-stratified analyses were not performed. While this balanced representation supports generalisability across sexes, potential differences in treatment response related to biological or gender-related factors cannot be excluded.

In addition, the intervention period was relatively short, implemented in recognition of the high-risk nature of the cohort, which precluded assessment of longer-term effects of AID on glycaemic, psychosocial and functional outcomes. Participants also received intensive clinical support during the study, which may have attenuated differences between treatment arms. The study population was predominantly white, which represents an additional limitation. As glycaemic patterns and comorbidity burden may vary across racial and ethnic groups, these findings may not be fully generalisable to more ethnically diverse populations. Finally, this study evaluated a single AID system, and the findings may not be generalisable to other AID technologies with differing algorithms or user interfaces.

Future research should aim to evaluate AID over longer durations to determine the sustainability of glycaemic benefits observed in the short term and whether benefits in qualitative outcomes emerge. Inclusion of a larger and more diverse cohort, particularly individuals receiving dialysis, will be important to assess the safety, usability and efficacy of AID in this complex subgroup. Optimising the interface of the AID system with the user, thereby minimising the physical and cognitive demands required to operate the technology, would facilitate implementation of these life-changing devices in this vulnerable cohort. Comparative studies incorporating multiple AID systems with differing algorithms and user interfaces are also warranted to determine whether outcomes are consistent across platforms and to identify features that optimise effectiveness and user satisfaction in people with advanced CKD.

These findings highlight the feasibility and tolerability of AID use in advanced CKD and underscore the importance of integrating both technological and behavioural strategies to optimise future diabetes care in this population.

## Supplementary Information

Below is the link to the electronic supplementary material.

ESM Table 1 (PDF 34 KB)

## Data Availability

Data are available from the corresponding author on reasonable request, subject to institutional data-sharing policies and ethical approval.

## Abbreviations

AID: Automated insulin delivery
CGM: Continuous glucose monitoring
CKD: Chronic kidney disease
DTSQs: Diabetes Treatment Satisfaction Questionnaire–Status
DTSQc: Diabetes Treatment Satisfaction Questionnaire–Change
EQ-5D: EuroQol-5 Dimension
ESKD: End stage kidney disease
GRI: Glycaemia Risk Index
HD: Haemodialysis
HFS-II-10: Short Form Hypoglycaemia Fear Survey-II
HREC: Human Research Ethics Committee
MDI: Multiple daily injections
MoCA: Montreal Cognitive Assessment
PAID: Problem Areas in Diabetes
PD: Peritoneal dialysis
PSQI: Pittsburgh Sleep Quality Index
SAE: Serious adverse event
SARC-F: Strength, Assistance in walking, Rising from a chair, Climbing stairs, and Falls
TIR: Time in range
