# Supplementary material for: The impact of automated insulin delivery on glucose management in people with diabetes and advanced chronic kidney disease
Source: Diabetologia. 2026 May 11;69(8):2168–81. doi: 10.1007/s00125-026-06732-3 (PMC13310223; doi:10.1007/s00125-026-06732-3)
Supplement: Supplementary file 1 — ESM Table 1 (PDF 34 KB) [file 125_2026_6732_MOESM1_ESM.pdf]

Supplementary Material

ESM Table 1: Inclusion exclusion criteria

**Key eligibility criteria:**	• Age 18–75 years<br>• Type 1 diabetes of at least 1-year duration or insulin requiring type 2 diabetes<br>• HbA1c <91 mmol/mol (10.5%)<br>• Managed with multiple daily injections (MDI [ie separate rapid acting and basal insulin injections, or $\geq$2 mixed insulin injections daily]) or insulin pump therapy (IPT)<br>• Renal function meets criteria: eGFR<45mL/min/1.73m^2, ESKD on peritoneal dialysis or ESKD on hemodialysis)<br>• Total daily dose of insulin <200 Units<br>• Participant (and carer where applicable) should be able to speak and understand English (at Australian sites) or Danish (at Danish site)<br>• Willing and able to implement the study requirements.<br>• Have internet or mobile phone access enabling upload of the AID system data
**Key exclusion criteria:**	• Sulphonylureas are not permitted. Metformin, SGLT2inh and GLP-1 analogues permitted within regulatory guidelines (pre-dialysis).<br>• Systemic glucocorticoid therapy within the last 4weeks (stable doses [>8 weeks] are permitted)<br>• DKA within past 4-weeks<br>• Pregnancy or planned pregnancy<br>• Major allergy to tape/ adhesives<br>• Active major life- threatening illness limiting life-expectancy to <6 months<br>• Major psychiatric history.
